# A Clinical Practice‐Based Comparison of Conventional and Individualized Dosing Strategies for Therapeutic Enoxaparin

**DOI:** 10.1002/prp2.70039

**Published:** 2025-01-24

**Authors:** Anthony Damiani, Viviane De Menezes Caceres, Greg Roberts, Jessica Coddo, Nicholas Scarfo, Desmond B. Willliams, Vinosshini Tharmathurai, Rami Tadros, Stephen Fitzgerald, Alice O'Connell, Amrit Kaur Sandhu, Andrew Vanlint, Arduino A. Mangoni, Dirk Hofmann, Hosam Bony, Jeff Faunt, Jir Ping Boey, Nicholas Farinola, Rachel Wells, Stephen Hedger, Udul Hewage, Yogesh Sharma, Zuhair Jabbar, Josephine Thomas, Katerina Flabouris, Toby Gilbert, Campbell Thompson, Patrick Russell

**Affiliations:** ^1^ SA Pharmacy Central Adelaide Local Health Network Adelaide South Australia Australia; ^2^ School of Allied Health Science and Practice The University of Adelaide Adelaide South Australia Australia; ^3^ SA Pharmacy Southern Adelaide Local Health Network Adelaide South Australia Australia; ^4^ Pharmacy and Biomedical Sciences University of South Australia Adelaide South Australia Australia; ^5^ Department of Internal Medicine Royal Adelaide Hospital, Central Adelaide Local Health Network Adelaide South Australia Australia; ^6^ Medical Services Division Northern Adelaide Local Health Network Adelaide South Australia Australia; ^7^ Department of Clinical Pharmacology Flinders University and Flinders Medical Centre Adelaide South Australia Australia; ^8^ Department of General Internal Medicine Noarlunga Hospital, Southern Adelaide Local Health Network Adelaide South Australia Australia; ^9^ Department of Internal Medicine Flinders Medical Centre Southern Adelaide Local Health Network Adelaide South Australia Australia; ^10^ Department of Clinical Pharmacology Royal Adelaide Hospital, Central Adelaide Local Health Network Adelaide South Australia Australia; ^11^ College of Medicine & Public Health Flinders University Adelaide South Australia Australia; ^12^ Discipline of Medicine The University of Adelaide Adelaide South Australia Australia

**Keywords:** enoxaparin, factor Xa, low‐molecular‐weight heparin

## Abstract

To understand differences in anti‐factor‐Xa levels produced by two different dosing strategies (conventional and individualized) for therapeutic enoxaparin in a cohort of hospital inpatients. A multicenter, retrospective cohort study over a two‐ and a half‐year period for inpatients with stable renal function and on therapeutic enoxaparin. Anti‐factor‐Xa levels were taken 3–5 h after enoxaparin administration and a minimum of 48 h of dosing. The final analysis included 278 patients from five hospitals: conventional dosing was used for 141, while 137 were given an unconventional dose, that is, individualized for their renal function and weight. Out‐of‐range levels were frequent (35% to 40% of all inpatients). After adjustment for age, renal function, and body mass index (BMI), the conventional group was more likely to experience above‐range levels (> 1.0 IU/mL; OR 2.50 [95% CI 1.38–4.56], *p* < 0.003) than the individualized group. Individualized dosing was independently associated with higher odds of a below‐range anti‐Xa level (< 0.5 IU/mL) compared to conventional dosing (OR 2.27 [95% CI 1.07–4.76], *p* = 0.03). Within the conventional group, above‐range levels were significantly and independently associated with decreasing renal function (OR 0.97, 95% CI 0.96–0.99, *p* = 0.004) and with increasing BMI (OR 1.06, 95% CI 1.01–1.10, *p* = 0.02). No such associations were seen with an individualized approach. Clinical event rates were low and not different between groups (*p* > 0.24). Conventional therapeutic dosing of enoxaparin exposed people with obesity or renal impairment to more frequent above‐range anti‐factor‐Xa levels; individualizing the dose could improve this but might expose people to subtherapeutic levels. More research is needed.

## Graphical abstract

Odds ratios for supratherapeutic anti‐Xa levels using conventional vs. individualised dosing of enoxaparin in the setting of worsening renal function or increasing obesity.

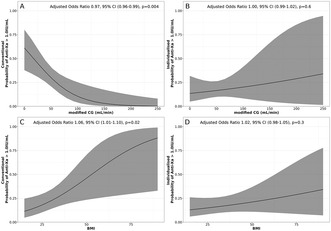

## 1 Introduction

Enoxaparin is a well‐recognized anticoagulant administered subcutaneously primarily in the hospital setting. Enoxaparin is prescribed most commonly for prophylaxis against venous thromboembolism (VTE), but is prescribed at higher, therapeutic doses for treatment of VTE, acute coronary syndrome, and bridging to oral therapy for atrial fibrillation or patients with mechanical heart valves.

The therapeutic dosing of enoxaparin is guided by the product information which recommends administering 1 mg kg^−1^ of actual body weight subcutaneously every 12 h (h) or 1.5 mg kg^−1^ subcutaneously every 24 h [1]. Enoxaparin relies on renal clearance for its elimination. When a patient's creatinine clearance (Cl_Cr_) is below 30 mL/min, the manufacturer recommends a 50% reduction in the dose [2]. But as renal function decreases from normal, long before reaching a Cl_Cr_ of 30 mL/min, a given dose of enoxaparin will produce a less predictable anticoagulant effect, measured as anti‐factor Xa concentration (anti‐Xa) [3]. In some older people, particularly those with Cl_Cr_ below 50 mL/min [4], biochemical estimates of renal function such as serum creatinine or the estimated glomerular filtration rate (GFR) automatically generated on pathology reports can overestimate renal function. Conventional dosing could lead to greater anticoagulant exposure than intended.

Enoxaparin affects the activity of clotting factor Xa. Measuring the level of anticoagulation with enoxaparin is performed using a chromogenic method so that the anti‐Xa level is proportional to the level of enoxaparin [5].

Obesity presents additional challenges as the product information does not suggest a fixed maximum daily dose (i.e., capped dose), and enoxaparin pharmacokinetics are impacted by obesity. There is conflicting advice in the literature regarding whether or not one should adjust dosing to accommodate extremes of weight. Although the 2018 guidelines of the American Hematology Association (AHA) advises using a patient's actual body weight without a fixed maximum dose, some evidence suggests otherwise for patients with obesity [6]. Indeed, a more recent systematic review supports a dose less than actual body weight [7]. The AHA further advises against using anti‐Xa concentration monitoring to guide dose adjustment for either patients with obesity or those with severe renal dysfunction (Cl_Cr_ < 30 mL/min) [8]. Studies reviewed for the guidelines found approximately 50% of patients with obesity treated with therapeutic doses (1 mg kg^−1^ actual body weight) of enoxaparin were within anti‐Xa level target range, but that adjustment of dosing based on anti‐Xa levels could be associated with increased risk of recurrent pulmonary embolism; confidence intervals of the point estimates referenced in the 2018 guidelines were extremely wide (relative risk, 3.06 [95% CI, 0.19–48.27]).

Evidence suggests a different approach to dosing can help more patients achieve an anti‐Xa level within the target range (0.5–1.0 IU/mL). Reducing the dose in line with reductions in renal function, beginning with a Cl_Cr_ 80 mL/min and in 10 mg decrements for every 10 mL/min reduction in Cl_Cr_, might reduce the frequency of observation of patients with elevated anti‐Xa levels and hence lower the associated clinical risk. We sought to understand whether conventional or individualized dosing of enoxaparin leads to more frequent initial anti‐Xa levels within the target range, with a primary concern of avoiding levels above the target range. Our null hypothesis was that there was no difference between the initial anti‐Xa levels produced by conventional dosing and the initial anti‐Xa levels produced by individualized dosing.

## 2 Methods

### 2.1 Study Population

This retrospective cohort study examined all anti‐Xa levels available for those receiving therapeutic enoxaparin admitted to five tertiary care hospitals in the two‐and‐a‐half‐year period between January 2019 and August 2021.

All admissions associated with anti‐Xa levels > 0.3 IU/mL along with corresponding medical record numbers were retrieved from the state‐wide pathology laboratory. We reviewed individual patient case notes, either paper or electronic, dependent on the admission site.

Subjects were included if aged > 18 years, prescribed therapeutic enoxaparin during admission, and assessed for anti‐Xa levels taken between 3 and 5 h post‐dose after a minimum of 48 h of enoxaparin therapy. Only the first eligible level from a patient's admission was included.

Subjects were excluded from the study if they met any of the following criteria: pregnant or breastfeeding; absence of weight, height or medical record; serum creatinine unavailable within 24 h of the anti‐Xa level; prescribed prophylactic enoxaparin; the dose of enoxaparin associated with the anti‐Xa level was administered in the outpatient setting; or if experiencing an acute kidney injury, defined as a serum creatinine increase of ≥ 26.5 μmol/L within 48 h or ≥ 1.5 × baseline within 7 days.

After applying inclusion and exclusion criteria to the patients whose anti‐Xa levels featured in the dataset, we reviewed the clinical notes at the time of the level, including the associated dose of enoxaparin. We backtracked each level to the dose that produced it and used patient weight and renal function to categorize each level as either “conventional” or “individualized”.

### 2.2 Definitions

#### 2.2.1 Dosing

The choice of once or twice‐daily dosing remained at the discretion of the prescriber. Conventional dosing (conventional group) was defined as the dose recommended by the manufacturer's product information (PI) of 1 mg kg^−1^ via subcutaneous injection twice‐daily or 1.5 mg kg^−1^ once‐daily for renal function above Cl_Cr_ 30 mL/min. Renal function was calculated using the modified Cockcroft‐Gault (CG) formula [9], Cl_Cr_ = {((140–age) × weight)/(72xS_Cr_)} × 0.85 (if female). If renal function was impaired to a Cl_Cr_ of < 30 mL/min, the manufacturer recommends the dose be reduced to 1 mg kg^−1^ once‐daily (Product information, Clexane); any dose delivered that was within 10% of this recommendation was considered conventionally dosed.

Any dose delivered that was greater than a 10% variation from conventional dosing, higher or lower than a conventional dose, was considered an individualized dose (individualized group). A specific individualized dosing strategy was recommended at one hospital, based on the findings of Green et al. [10], and using adjustments for body weight and renal function.

Renally adjusted doses in that strategy (in mg kg^−1^) were as follows:

- Cl_Cr_ 70–79 mL/min: 0.9 mg kg^−1^ administered twice‐daily
- Cl_Cr_ 60–69 mL/min: 0.8 mg kg^−1^ administered twice‐daily
- Cl_Cr_ 50–59 mL/min: 0.7 mg kg^−1^ administered twice‐daily
- Cl_Cr_ 40–49 mL/min: 0.6 mg kg^−1^ administered twice‐daily
- Cl_Cr_ < 40 mL/min: 0.5 mg kg^−1^ administered twice‐daily

The twice‐daily dosing strategies—individualized and conventional—were the same for those patients with a Cl_Cr_ < 30 mL/min except that for individualized dosing a single stat dose of 1 mg kg^−1^ was recommended as the first dose for patients with Cl_Cr_ < 50 mL/min and continued at the renally adjusted dose thereafter. As part of the recommended invidualized strategy, actual weight was utilized to calculate doses with a maximum weight capped at 30% above the ideal body weight. Only the individualized group had dosing weight capped for obesity. The conventional group used actual body weight with no weight cap.

Sometimes doses appeared individualized for other reasons: a dose being delivered that was different from the dose prescribed (by only a few mg), the patient reported a weight different from their actual weight or the prescriber guessed the patient's body weight and dosed accordingly.

Doses greater than or equal to a 10% reduction from 1 mg kg^−1^ twice‐daily or 1.5 mg kg^−1^ daily were considered individualized since the individualized strategy of Green, et al., directs a dose reduction in 10% decrements.

We considered the dose preceding the anti‐Xa level to be stable if the dose was unchanged in the 48 h prior to the anti‐Xa level. We used the weight closest to the dose and analyzed by body mass index (BMI) to adjust for height.

### 2.3 Assay Methods and Levels

Anti‐Xa assay was performed at a single central laboratory. The anti‐Xa level was performed in citrate plasma using a chromogenic assay on a Stago instrument using STA‐liquid Anti‐Xa. The detection threshold was 0.10 IU/mL for low molecular weight heparin.

We used an anti‐Xa target range of 0.5–1.0 IU/mL for twice‐daily dosing.

For patients with a CrCl > 30 mL/min given *once‐daily* enoxaparin at a dose of 1.5 mg kg^−1^ (conventional dosing), we used the laboratory‐defined anti‐Xa target range of 1.0–2.0 IU/mL. For patients with a CrCl < 30 mL/min given *once‐daily* enoxaparin at 1 mg kg^−1^ (conventional dosing), we used a laboratory‐defined anti‐Xa target range of 0.5–1.0 IU/mL.

For patients with a CrCl < 30 mL/min given once‐daily enoxaparin at a dose < 1 mg kg^−1^ (individualized dose), we used an anti‐Xa target range of 0.5–1.0 IU/mL. For patients with a CrCl > 30 mL/min given once‐daily at a dose < 1.5 mg kg^−1^ (individualized dose), we used the anti‐Xa target range of 1.0–2.0 IU/mL.

Research Electronic Data Capture (REDCap) was used to collect clinical data of interest and classified into two domains: medical and pharmacy, entered by doctors or clinical pharmacists, respectively. The study was approved by the Central Adelaide Local Health Network (CAHLN) Human Research Ethics Committee (reference number 15357).

### 2.4 Outcomes and Data Analysis

The primary outcome of interest was an anti‐Xa level that was above range, defined at our state‐wide laboratory as anti‐Xa > 1.0 IU/mL for twice‐daily dosing, or > 2.0 IU/mL for once‐daily dosing, taken 3–5 h post‐dose. Multivariable logistic regression was used to assess the relationship between the main outcome and dosing approach, BMI, age, and renal function. Secondary outcomes included comparison of anti‐Xa levels below range (< 0.5 IU/mL) and mean anti‐Xa levels between groups. Separate analysis was performed for subgroups of interest: BMI < 30 kg/m^2^, 30–40 kg/m^2^, and ≥ 40 kg/m^2^; creatinine clearance < 50 mL/min, 30–49.9 mL/min and ≥ 50 mL/min; and age < 65 years and ≥ 65 years. These were compared both within and across the two study groups using a Student's *t*‐test for continuous anti‐Xa data and Chi‐square or Fisher's exact for categorical data. Statistical significance was defined as < 0.05 for the *p*‐value.

Demographic data were categorized by age, gender, height, weight, and renal function. Other captured data fields included indication for enoxaparin, concurrent medications, in‐hospital major bleeding using an accepted definition [11], and in‐hospital mortality.

## 3 Results

**FIGURE 1:**
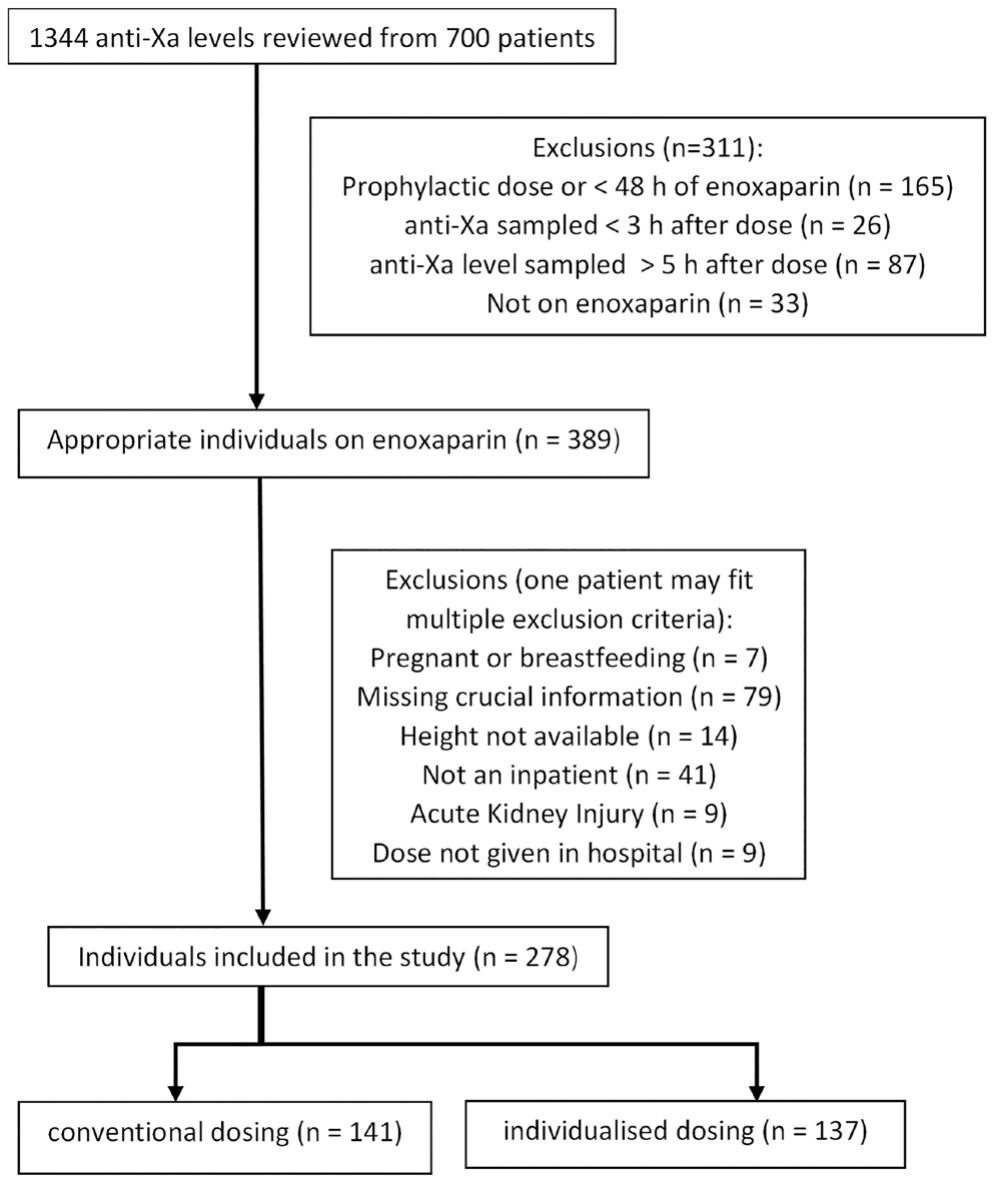
Patient recruitment.

We reviewed the medical records relating to 1344 anti‐Xa levels taken from 700 patients over the study period. After applying exclusion criteria, there were 278 subjects eligible for analysis, 141 in the conventional group and 137 in the individualized group (Figure 1). Baseline characteristics of patients were similar between the two groups apart from body weight, which was significantly lower in the conventional group. Patients in the conventional group also had better renal function when compared to those in the individualized group (Table 1).

**TABLE 1:** Characteristics of conventional dosing and individualized dosing groups. Mean (SD) unless otherwise stated.

Characteristics		Conventional dosing (*n* = 141)	Individualized dosing (*n* = 137)	*p*
Age (years)		65.4 (15.1)	68.8 (14.6)	0.06
Age ≥ 65 years, *N* (%)		81 (57.4)	87 (63.5)	0.36
Age < 65 years, *N* (%)		60 (42.6)	50 (36.5)	
Male, *N* (%)		90 (63.8)	82 (59.9)	0.58
Length of stay (days), median (IQR)		12.0 (6.0–24.0)	14.0 (8.0–25.0)	0.18
Height (cm)		171.2 (10.3)	170.5 (10.4)	0.55
Weight (kg)		88.02 (26.9)	98.32 (41.0)	0.01
BMI (kg/m^2^), median (IQR)		28.9 (24.2–33.7)	29.5 (25.0–39.7)	0.13
BMI ≥ 30 kg/m^2^, *N* (%)		64 (45.40)	64 (46.70)	0.92
Estimated GFR (mL/min/1.73m^2^)		50.4 (27.9)	48.33 (23.2)	0.60
≥ 50, *N* (%)		43 (30.5)	40 (29.2)	0.01
30–50, *N* (%)		15 (10.6)	37 (27.0)	
< 30, *N* (%)		25 (17.7)	21 (15.3)	
CrCl (modified CG, mL/min)		65.3 (36.3)	55.3 (33.3)	0.02
≥ 50, *N* (%)		89 (63.1)	61 (44.5)	0.002
30–50, *N* (%)		26 (18.4)	49 (35.8)	
< 30, *N* (%)		26 (18.4)	27 (19.7)	
Anti‐Xa level (IU/mL)		0.88 (0.35)	0.79 (0.32)	0.02
Anti‐Xa > 1.0 IU/mL *N* (%)		45 (31.9)	26 (19.0)	0.02
CrCl^a^		0.97 (0.96, 0.99)	1.00 (0.99, 1.02)	
Age^a^		0.97 (0.94, 1.00)	1.02 (0.98, 1.06)	
Dosing frequency (twice‐daily)		3.72 (1.06–13.01)	1.46 (0.44, 4.84)	
BMI^a^		1.06 (1.01, 1.10)	1.02 (0.98, 1.05)	
Anti‐Xa < 0.5 IU/mL *N* (%)		13 (9.2)	23 (16.8)	0.09
Enoxaparin dose (mg)		88.2 (25.5)	77.6 (32.8)	0.003
Enoxaparin dose (mg kg^−1^/dose)		1.01 (0.12)	0.83 (0.30)	< 0.0001
Once‐daily dosing, *N* (%)		29 (20.6)	29 (21.2)	1.00
Concurrent antiplatelet treatment, *N* (%)		1 (0.70)	1 (0.70)	1.00
Clinical events, *N* (%)				
Death		12 (8.50)	10 (7.30)	0.88
Acute coronary syndrome		0 (0.0)	1 (0.7)	0.49
Venous thromboembolism		2 (1.40)	2 (1.50)	1.00
Ischaemic stroke		0 (0.0)	2 (1.50)	0.24
Major bleeding		5 (3.50)	8 (5.80)	0.53
Indication(s) for enoxaparin, *N* (%)				
Pulmonary embolism		85 (60.30)	72 (52.60)	0.24
Deep vein thrombosis		70 (49.60)	52 (38.0)	0.07
Atrial fibrillation		24 (17.0)	30 (21.90)	0.38
Myocardial infarction		1 (0.70)	2 (1.50)	0.62
Mechanical heart valve		5 (3.50)	7 (5.10)	0.73
Other/unknown		1 (0.70)	0 (0.0)	1.00

Abbreviations: BMI = body mass index, CG = Cockcroft Gault, CKD = chronic kidney disease, CrCl = creatinine clearance (modified), GFR = glomerular filtration rate, IU = international units, SD = standard deviation.

^a^ Odds ratio (95% CI) from subgroup analysis within conventional and individualized group.

More than one‐third of anti‐Xa levels were out of range, above or below the laboratory‐defined target (Table 2).

**TABLE 2:** Comparison of individualized and conventional subgroups of interest for anti Xa > 1.0 IU/mL, anti Xa < 0.5 IU/mL, and anti Xa (IU/mL).

	Conventional group		Individualized group		*p*
Anti‐Xa > 1.0 IU/mL *N* (%)	Total^a^	*N* (%)^b^ > 1.0 IU/mL	Total^a^	*N* (%)^b^ > 1.0 IU/mL	
Cl_Cr_ < 50 mL/min	52	19 (36.5)	76	12 (15.8)	0.0111
Cl_Cr_ ≥ 50 mL/min	89	26 (29.2)	61	14 (23.0)	0.45
Cl_Cr_ 30–50 mL/min	26	11 (42.3)	49	8 (16.3)	0.0241
BMI < 30	77	16 (20.8)	73	15 (20.5)	1.00
BMI ≥ 30	64	29 (45.3)	64	11 (17.2)	0.0010
BMI ≥ 30 and < 40	51	22 (43.1)	31	4 (12.9)	0.0065
< 65 years	60	18 (30.0)	50	8 (16.0)	0.11
≥ 65 years	81	27 (33.3)	87	18 (20.7)	0.0814
Anti‐Xa < 0.5 IU/mL *N* (%)	Total^a^	*N* (%)^c^ < 0.5 IU/mL	Total^a^	*N* (%)^c^ < 0.5 IU/mL	
Cl_Cr_ < 50 mL/min	52	5 (9.6)	76	16 (21.1)	0.0957
Cl_Cr_ ≥ 50 mL/min	89	8 (9.0)	61	7 (11.5)	0.82
Cl_Cr_ 30–50 mL/min	26	1 (3.8)	49	11 (22.4)	0.0477
BMI < 30	77	8 (10.4)	73	15 (20.5)	0.1125
BMI ≥ 30	64	5 (7.8)	64	8 (12.5)	0.56
BMI ≥ 30 and < 40	51	4 (7.8)	31	7 (22.6)	0.0924
< 65 years	60	4 (6.7)	50	5 (10.0)	0.73
≥ 65 years	81	9 (11.1)	87	18 (20.7)	0.0981
Anti‐Xa (IU/mL)	Total^a^	Mean ± SD	Total^a^	Mean ± SD	
Cl_Cr_ < 50 mL/min	52	0.93 ± 0.38	76	0.75 ± 0.33	0.009
Cl_Cr_ ≥ 50 mL/min	89	0.86 ± 0.33	61	0.84 ± 0.31	0.74
Cl_Cr_ 30–50 mL/min	26	1.02 + 0.42	49	0.75 + 0.31	0.006
BMI < 30	77	0.81 ± 0.31	73	0.78 ± 0.32	0.59
BMI ≥ 30	64	0.97 ± 0.38	64	0.80 ± 0.33	0.0084
BMI ≥ 30 and < 40	51	0.95 + 0.35	31	0.80 + 0.38	0.0567
< 65 years	60	0.85 ± 0.31	50	0.82 ± 0.30	0.57
≥ 65 years	81	0.91 ± 0.37	87	0.78 ± 0.34	0.0185

^a^ Total number of patients in conventional or individualized group within each subgroup of interest.

^b^ Percentages are number of above range anti‐Xa levels out of the total number within each subgroup of interest.

^c^ Percentages are number of below range anti‐Xa levels out of the total number within each subgroup of interest.

### 3.1 Above‐Range Levels (Anti‐Xa > 1.0 IU/mL)

Conventional dosing was statistically significantly associated with higher odds of an above range anti‐Xa level compared to individualized dosing. After adjustment for age, renal function, BMI, and dosing frequency (once‐ vs. twice‐daily), patients whose anti‐Xa levels were measured after receiving conventional dosing had a greater odds of being above range (odds ratio (OR) 2.50, 95% CI 1.37–4.56, *p* = 0.003) than patients whose anti‐Xa levels were measured after receiving a dose from the individualized strategy (Table 3).

**TABLE 3:** Multivariate relationships with above‐range anti‐Xa (> 1.0 IU/mL) for all patients, conventional dose group and individualized group.

	Odds ratio	95% CI	*p*
All patients (*n* = 278)			
Dosing frequency	1.99	0.86–4.59	0.10
Age (years)	0.99	0.97–1.02	0.49
GFR (mod CG)	0.989	0.977–1.001	0.0606
BMI	1.026	1.000–1.051	0.0485
Study group	2.50	1.38–4.56	0.0027
Conventional group (*n* = 141)			
Dosing frequency	3.72	1.06–13.01	0.04
Age (years)	0.97	0.97–1.00	0.09
GFR (mod CG)	0.97	0.96–0.99	0.004
BMI	1.06	1.01–1.10	0.02
Individualized group (*n* = 137)			
Dosing frequency	1.46	0.44–4.84	0.53
Age (years)	1.02	0.98–1.06	0.35
GFR (mod CG)	1.00	0.99–1.02	0.60
BMI	1.02	0.98–1.05	0.32

**FIGURE 2:**
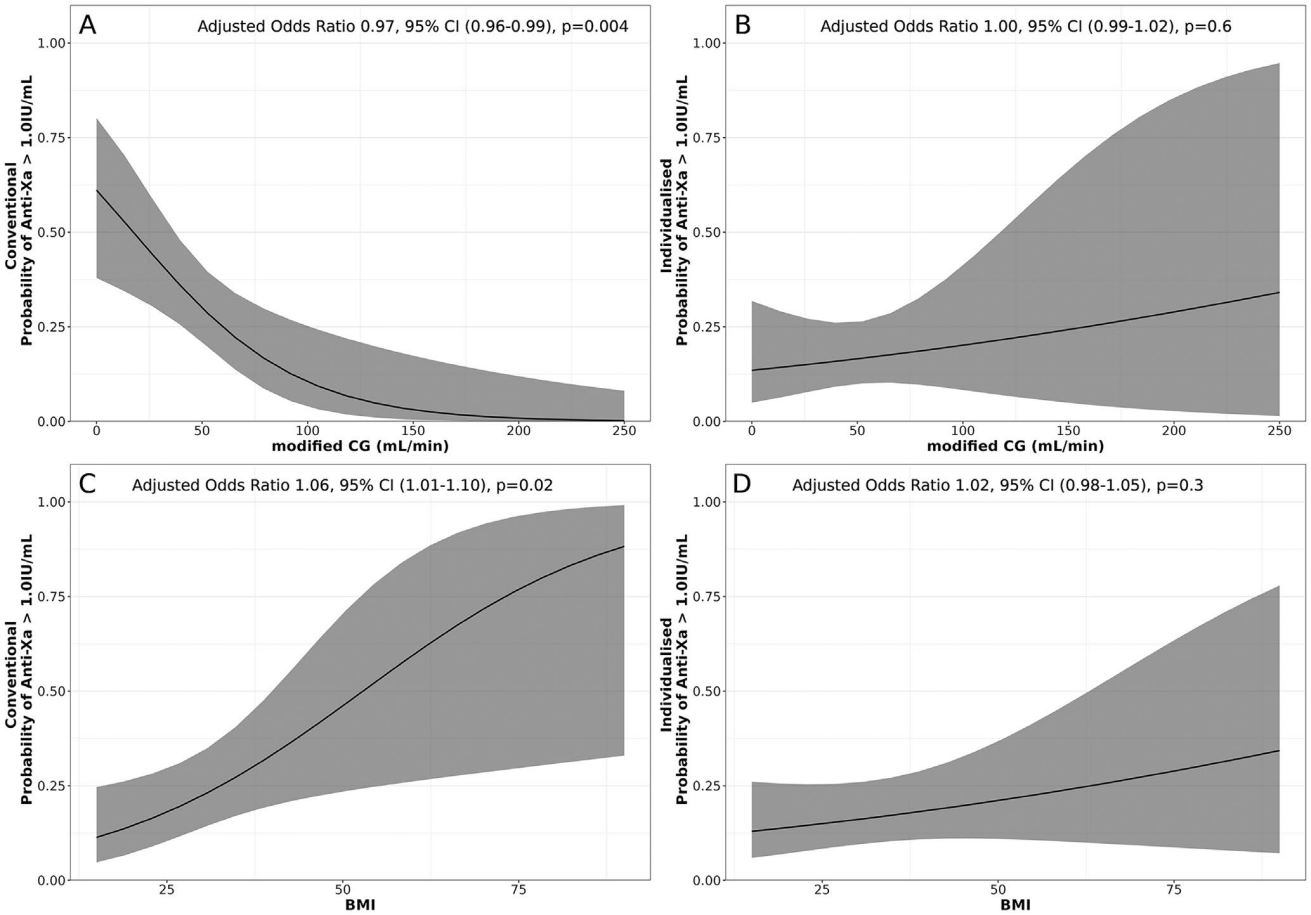
Likelihood of an anti‐Xa level > 1.0 IU/mL within conventional and individualized groups for body mass index: (A, B) creatinine clearance. (C, D) BMI.

Within the conventional group, renal function remained independently associated with the odds of a patient's anti‐Xa level being over 1.0 IU/mL (OR 0.97, 95% CI 0.96–0.99, *p* = 0.004) (Figure 2). Conversely, within the individualized group cohort, there was no independent association between Cl_Cr_ and above‐range levels of anti‐Xa, nor was there any similar association of above‐range levels of anti‐Xa with BMI.

The greater a patient's BMI, the greater were the odds of an above range anti‐Xa level occurring (Figure 2). This was a small but statistically significant association, independent of patients' age, GFR, and dosing frequency, with an estimated increase of anti‐Xa level of 3% for every 1 kg/m^2^ increase in BMI (OR 1.03, 95% CI 1.00–1.05, *p* = 0.0485).

Age was not independently associated with an above range anti‐Xa level in either group.

### 3.2 Below‐Range Levels (Anti‐Xa < 0.5 IU/mL)

Individualized dosing was independently associated with higher odds of a below‐range anti‐Xa level compared to conventional dosing (OR = 2.27 [95% CI 1.07, 4.76] *p* = 0.03). Within group analysis of conventional and individualized cohorts showed no independent association of any variables of interest with below‐range anti‐Xa. Once‐daily dosing, an option prescribers chose 58 times overall, did not affect the odds of a below‐range anti‐Xa level.

In subgroup analysis, the addition of study site as a variable did not impact outcomes. In further subgroup analysis of only those receiving twice‐daily dosing (*n* = 220), all of the above relationships were maintained (Table S1).

The adjusted probability of an anti‐Xa above 1.0 IU/m for the conventional and individualized groups comparing the impact of creatinine clearance (Figure 2A,B) and BMI (Figure 2C,D) are presented graphically. This highlights the marked increase in probability of above‐range anti‐Xa as creatinine clearance decreases for the conventional group with little change across the range of creatinine clearance in the individualized group (Figure 2A,B). Likewise, there is a marked increase in the probability of above‐range anti‐Xa as BMI increases for those dosed conventionally with little change with increasing BMI for the individualized group (Figure 2C,D).

## 4 Discussion

Optimal use of anticoagulants requires clarity about risk. In this study, we examined all anti‐factor Xa levels sent to our state‐wide pathology service from five hospitals across a two‐and‐a‐half‐year period. We traced these levels back to those patients taking therapeutic enoxaparin to ascertain a difference between the initial levels produced by conventional dosing and the levels produced by a strategy that is more tailored to an individual's renal function and weight. What we found enlightened us. We found the strongest relationships with anti‐Xa levels and study group or BMI (Table 4).

**TABLE 4:** Multivariable relationships with anti‐Xa levels (as a continuous variable) for all patients.

	Odds ratio (95% CI)	*p*
Dosing frequency (twice‐daily)	1.08 (0.95–1.23)	0.2
Age (years)	1.01 (0.99–1.01)	0.8
GFR (mod CG)	0.99 (0.99–1.01)	0.5
BMI	1.005 (1.000–1.009)	0.04
Study group (conventional group)	1.15 (1.04–1.26)	0.005

*First*, the dosing strategy had an effect on anti‐Xa levels; the authors rejected the null hypothesis. Patients with stable moderate renal impairment, not severe enough for a conventional dose reduction, had a greater odds of an anti‐factor Xa concentration that was above the target range when dosed according to the manufacturer's PI. This makes sense. Why would someone with a Cl_Cr_ of 31 mL/min be given twice the dose of someone with a Cl_Cr_ of 29 mL/min? We argue that convention has most defined this practice: the dose adjustment for severe renal dysfunction that came after the Thrombolysis In Myocardial Infarction (TIMI) 11b and the Efficacy Safety Subcutaenous Enoxaparin in Non‐Q‐wave Coronary Events (ESSENCE) studies [12] clarified safety; first in, best dressed. But a better alternative to the PI might be available. Indeed, in a survey of hospitals in Australia, New Zealand, United States, and the United Kingdom, of over 250 surveys suitable for analysis, 96% preferred a dosing guide other than the product label [13].

Patients dosed more precisely, using dose reductions that more closely mirror reductions in renal function, also had a greater odds of a below range anti‐Xa level. Loading doses (1 mg kg^−1^ body weight) were not recorded during the data collection. We considered the exclusion of levels taken before Day 3 of treatment would sufficiently address the issue of a loading dose, but now recognize that some patients with chronic kidney disease might not reach steady state for several days more. Anti‐Xa levels after 48 h of conventional dosing for a patient with CKD, on the other hand, would essentially be equivalent to those taken after a loading dose.

*Second*, both dosing strategies frequently produce out‐of‐range levels (35%–40%). Clinicians look and find above range levels of anti‐Xa, despite the American Hematology Association (AHA) 2018 (conditional) recommendation against using anti‐factor Xa concentration when monitoring patients with (*severe*) renal impairment during the treatment of VTE [14]. But it is a conditional recommendation of that association because the strength of the evidence is low. The link between major clinical events—hemorrhage or thrombosis—and out of range anti‐Xa levels—too high or too low—is challenged by the low incidence of these events. The clinical impact of anti‐Xa levels that are too low, compared to those which are too high, is difficult to ascertain against the backdrop of small, single‐site observational studies underpinning the AHA's recommendation.

Most of the literature that fails to show a relationship between anti‐factor Xa levels and clinical events comes from studies of prophylactic, not therapeutic, dosing of enoxaparin. While it is important to know that prophylactic doses are unlikely to result in hemorrhage and that an anti‐factor Xa level produced by a prophylactic dose will not help predict bleeding, it does little to quell worry at the bedside when a patient of short stature and weighing > 150 kg is prescribed a dose of 1 mg kg^−1^ using actual body weight and administered twice a day. As the prevalence of obesity in hospitalized patients continues to increase, so will this worry. We don't understand clinical impact of out‐of‐range anti‐Xa levels.

*Third*, weight matters. When the 64 obese patients were dosed according to the conventional strategy, the anti‐Xa levels produced were skewed toward above‐range risk. Each increase in BMI of 10 kg/m^2^ was independently associated with a 28.6% (95% CI 0.2%–65.1%) increased odds of an above‐range anti‐Xa level. We think this predictably higher occurrence of above‐range levels is unnecessary and deserves greater research attention, especially in light of the ever‐increasing prevalence of obesity and morbid obesity in hospitals around the globe. Other studies of anti‐Xa levels in obese patients published since 2018 have raised concern about unadjusted dosing of enoxaparin for patients with obesity [15, 16]. It is clear more data are needed.

*Fourth*, age made no difference. Those people above 65 years were not more likely to experience an above‐range anti‐Xa level than younger patients after receiving a therapeutic dose of enoxaparin. This is in line with other clinical scenarios where robust older people behave physiologically like younger patients (e.g., intensive control of blood pressure). Age, by itself, was not associated with above range levels of anti‐factor Xa. This finding will help us to develop a protocol for a randomized trial that will focus on the right questions.

*Fifth*, once‐daily dosing affected the mean anti‐Xa levels, but not the frequency of above‐ or below‐range levels. Once‐daily dosing was included because it is a frequent choice made and a part of the real‐world clinical practice.

*Lastly*, the incidence of significant clinical adverse events was low. This is consistent with previous literature where hemorrhage was often < 1% and thrombosis < 5% while on therapeutic dosing even without knowledge of anti‐Xa levels [14, 17]. This also means any RCT powered to detect differences in hemorrhage or recurrent thrombosis, rather than appropriate levels of anti‐Xa levels, could require many thousands of participants and many sites.

### 4.1 For the Future

The differences we noted in anti‐Xa levels associated with dosing strategies give thrust to those clinicians committed to the available clinical decision support tools that make dose calculations easier for busy prescribers—easier to inform patients and easier to tailor therapy. One size rarely fits all. But we can see that more research is needed. This study leaves room for a randomized clinical trial comparing conventional dosing (e.g., 1 mg kg^−1^ twice‐daily) for patients with a Cl_Cr_ 30–50 mL/min to a dosing strategy more aligned with the first‐order kinetics of enoxaparin. Using within‐range anti‐Xa levels as a primary outcome, our data indicate a sample size of at least 301 per group (*n* = 602 in total) to achieve 90% power, with an alpha level of 0.05 and assuming a 7% difference in anti‐Xa levels between the groups.

Alternatively, a large observational study of anti‐Xa levels and significant clinical events could help clinicians understand better the role of anti‐Xa monitoring, the risks attending levels too high or low, and the economic impact of monitoring in order to prevent one significant clinical event.

## 5 Strengths and Limitations

The strength of this study is the large, multicenter group of patients commonly encountered on a busy hospital ward and prescribed enoxaparin using different dosing strategies by busy clinicians. This “real‐life” environment increases the generalizability of our results. In our study, 128 (46%) patients were obese. Additionally, the tight definition of peak level and acute kidney injury helped reduce the influence of any fluctuation in renal function that could affect the anti‐Xa levels. But our study also carries significant limitations. The first is the selection bias: only people with anti‐factor Xa levels were included; second, there is no quantification of what proportion of inpatients on therapeutic enoxaparin this represents or understanding of why levels were sent in the first place. Third, there were not enough clinical events in either group to draw conclusions about the clinical impact of a level too high or one too low. We acknowledge that busy prescribers and pharmacists might be more interested in actual clinical events than risk.

While the individualized dosing used in this study might be safer for those with a high BMI or moderate renal impairment, there remains opportunity to further improve the individualized dosing. Use of an appropriate loading dose as described by others [18] could reduce exposure to below‐range levels during the first few days. Future research could also focus on one at‐risk group or another—moderate renal impairment or obesity—rather than both combined.

## 6 Conclusion

In conclusion, conventional dosing for therapeutic enoxaparin could expose patients to risks of over‐anticoagulation; tailoring the dose to a patient's renal function and BMI could reduce that risk. The lower dose associated with a more individualized approach could paradoxically expose a patient to the risk of under‐anticoagulation. More research is needed.

## Author Contributions

Patrick Russell, Anthony Damiani, Viviane De Menezes Caceres, Greg Roberts, Desmond B. Willliams, Toby Gilbert, Campbell Thompson, Jessica Coddo, Nicholas Scarfo, Vinosshini Tharmathurai, Hosam Bony, and Andrew Vanlint collected data, helped draft the manuscript, and approved the final version submitted. Nicholas Farinola, Rachel Wells, Stephen Hedger, Udul Hewage, Yogesh Sharma, Zuhair Jabbar, Josephine Thomas, Katerina Flabouris, Rami Tadros, Stephen Fitzgerald, Alice O'Connell, Amrit Kaur Sandhu, Arduino A. Mangoni, Dirk Hofmann, Jeff Faunt, and Jir Ping Boey played critical roles in planning and execution, helped draft the manuscript, and approved the final version submitted.

## Ethics Statement

The study was approved by the Central Adelaide Local Health Network (CAHLN) Human Research Ethics Committee (reference number 15357).

## Conflicts of Interest

The authors declare no conflicts of interest.

## Supporting information


**Table S1.** Multivariable relationships with above range anti‐Xa (> 1.0 IU/mL) for all patients using twice‐daily dosing only.

## Data Availability

The data that support the findings of this study are available from Electronic Medical Records (EMR) and patient case notes from SA Health (Central Adelaide Local Health Network and South Adelaide Local Health Network). Ethical restrictions apply to the availability of these data, which were used under request and license for this study. Data from Central Adelaide Local Health Network and South Adelaide Local Health Network can be made available within the data environment by application to and approval.

## References

[1] Sanofi‐Aventis U.S. LLC , “Lovenox (Enoxaparin Sodium Injection) Package Insert,” Bridgewater, NJ: Sanofi‐Aventis U.S. LLC; 2007. In: Laval, Quebec: sanofi‐aventis Canada Inc.

[2] R. Bruno , P. Baille , S. Retout , et al., “Population Pharmacokinetics and Pharmacodynamics of Enoxaparin in Unstable Angina and Non‐ST‐Segment Elevation Myocardial Infarction,” British Journal of Clinical Pharmacology 56 (2003): 407–414. 12968985 10.1046/j.1365-2125.2003.01904.x PMC1884380

[3] A. M. Frydman , L. Bara , Y. H. Roux , M. Woler , F. Chauliac , and M. M. Samama , “The Antithrombotic Activity and Pharmacokinetics of Enoxaparine, A Low Molecular Weight Heparin, in Humans Given Single Subcutaneous Doses of 20 to 80 Mg,” Journal of Clinical Pharmacology 28 (1988): 609–618. 2851016 10.1002/j.1552-4604.1988.tb03184.x

[4] G. W. Roberts , P. M. Ibsen , and C. T. Schioler , “Modified Diet in Renal Disease Method Overestimates Renal Function in Selected Elderly Patients,” Age and Ageing 38 (2009): 698–703. 19767628 10.1093/ageing/afp168

[5] M. Barras , “Anti‐Xa assays,” Australian Prescriber 36 (2013): 98–101.

[6] B. Green and S. B. Duffull , “Development of a Dosing Strategy for Enoxaparin in Obese Patients,” British Journal of Clinical Pharmacology 56 (2003): 96–103. 12848781 10.1046/j.1365-2125.2003.01849.x PMC1884333

[7] M. R. Chilbert , K. Zammit , U. Ahmed , et al., “A Systematic Review of Therapeutic Enoxaparin Dosing in Obesity,” Journal of Thrombosis and Thrombolysis 57 (2024): 587–597. 38402505 10.1007/s11239-024-02951-w

[8] D. M. Witt , R. Nieuwlaat , N. P. Clark , et al., “American Society of Hematology 2018 Guidelines for Management of Venous Thromboembolism: Optimal Management of Anticoagulation Therapy,” Blood Advances 2 (2018): 3257–3291. 30482765 10.1182/bloodadvances.2018024893 PMC6258922

[9] C. M. Kirkpatrick , S. B. Duffull , and E. J. Begg , “Pharmacokinetics of Gentamicin in 957 Patients With Varying Renal Function Dosed Once Daily,” British Journal of Clinical Pharmacology 47 (1999): 637–643. 10383541 10.1046/j.1365-2125.1999.00938.x PMC2014263

[10] B. Green , M. Greenwood , D. Saltissi , et al., “Dosing Strategy for Enoxaparin in Patients With Renal Impairment Presenting With Acute Coronary Syndromes,” British Journal of Clinical Pharmacology 59 (2005): 281–290. 15752373 10.1111/j.1365-2125.2004.02253.x PMC1884796

[11] S. Schulman and C. Kearon , “Definition of Major Bleeding in Clinical Investigations of Antihemostatic Medicinal Products in Non‐Surgical Patients,” Journal of Thrombosis and Haemostasis 3 (2005): 692–694. 15842354 10.1111/j.1538-7836.2005.01204.x

[12] S. A. Spinler , S. M. Inverso , M. Cohen , S. G. Goodman , K. A. Stringer , and E. M. Antman , “Safety and Efficacy of Unfractionated Heparin Versus Enoxaparin in Patients Who Are Obese and Patients With Severe Renal Impairment: Analysis From the ESSENCE and TIMI 11B Studies,” American Heart Journal 146 (2003): 33–41. 12851605 10.1016/S0002-8703(03)00121-2

[13] M. A. Barras , C. M. J. Kirkpatrick , and B. Green , “Current Dosing of Low‐Molecular‐Weight Heparins Does Not Reflect Licensed Product Labels: An International Survey,” British Journal of Clinical Pharmacology 69 (2010): 520–528. 20573088 10.1111/j.1365-2125.2010.03626.x PMC2856053

[14] P. M. Erkens and M. H. Prins , “Fixed Dose Subcutaneous Low Molecular Weight Heparins Versus Adjusted Dose Unfractionated Heparin for Venous Thromboembolism,” Cochrane Database of Systematic Reviews 9 (2010): CD001100. 10.1002/14651858.CD001100.pub3 20824828

[15] Y. R. Lee , P. J. Palmere , C. E. Burton , and T. M. Benavides , “Stratifying Therapeutic Enoxaparin Dose in Morbidly Obese Patients by BMI Class: A Retrospective Cohort Study,” Clinical Drug Investigation 40 (2020): 33–40. 31625111 10.1007/s40261-019-00855-9

[16] V. Sharma , L. Terrell , C. Karlapalem , V. Sermadevi , and F. Sadaka , “1020: Therapeutic Enoxaparin In Morbidly Obese Patients: Single‐Center Experience,” Critical Care Medicine 51 (2023): 504.

[17] L. R. Dolovich , J. S. Ginsberg , J. D. Douketis , A. M. Holbrook , and G. Cheah , “A Meta‐Analysis Comparing Low‐Molecular‐Weight Heparins With Unfractionated Heparin in the Treatment of Venous Thromboembolism: Examining Some Unanswered Questions Regarding Location of Treatment, Product Type, and Dosing Frequency,” Archives of Internal Medicine 160 (2000): 181. 10647756 10.1001/archinte.160.2.181

[18] J. Hulot , G. Montalescot , P. Lechat , J. Collet , A. Ankri , and S. Urien , “Dosing Strategy in Patients With Renal Failure Receiving Enoxaparin for the Treatment of Non‐ST‐Segment Elevation Acute Coronary Syndrome,” Clinical Pharmacology & Therapeutics 77 (2005): 542–552. 15961985 10.1016/j.clpt.2005.02.012

