# Supplementary figures and images for: A Clinical Practice‐Based Comparison of Conventional and Individualized Dosing Strategies for Therapeutic Enoxaparin
Source: Pharmacol Res Perspect. 2025 Jan 24;13(1):e70039. doi: 10.1002/prp2.70039 (PMC11760983; doi:10.1002/prp2.70039)

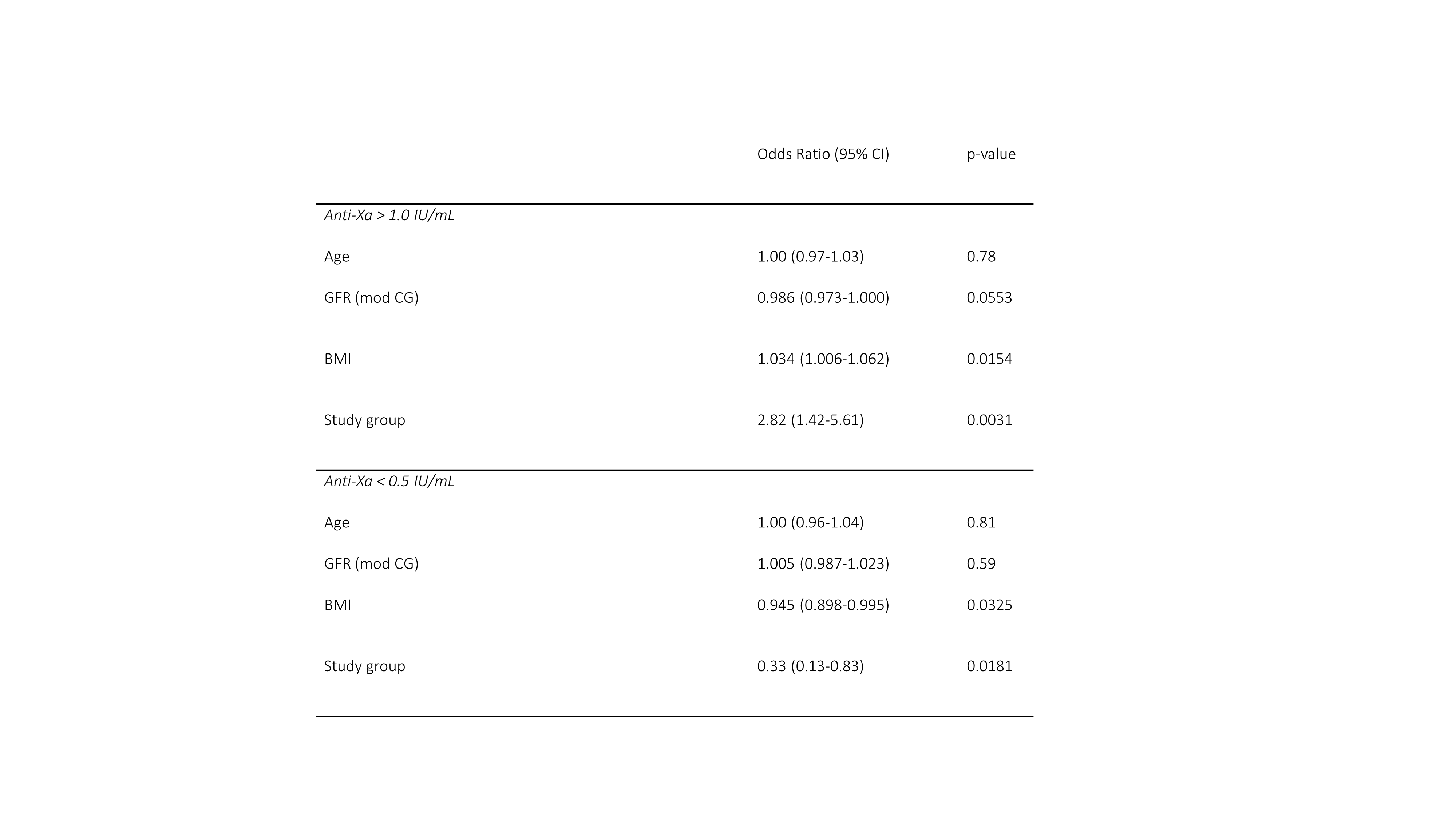

Supplement: Supplementary file 1 — Table S1. Multivariable relationships with above range anti‐Xa (> 1.0 IU/mL) for all patients using twice‐daily dosing only. [file PRP2-13-e70039-s001.tif]
